# Activity Patterns in Response to Symptoms in Patients Being Treated for Chronic Fatigue Syndrome: An Experience Sampling Methodology Study

**DOI:** 10.1037/hea0000422

**Published:** 2016-11-07

**Authors:** Rebecca Band, Christine Barrowclough, Kim Caldwell, Richard Emsley, Alison Wearden

**Affiliations:** 1 School of Psychological Sciences and Manchester Centre for Health Psychology, University of Manchester, and Centre for Applications of Health Psychology, University of Southampton; 2 School of Psychological Sciences and Manchester Centre for Health Psychology, University of Manchester; 3 Centre for Biostatistics, Institute of Population Health, University of Manchester; 4 School of Psychological Sciences and Manchester Centre for Health Psychology, University of Manchester

**Keywords:** chronic fatigue syndrome, experience sampling methodology, ecological momentary assessment, activity, behaviors

## Abstract

***Objective:*** Cognitive–behavioral models of chronic fatigue syndrome (CFS) propose that patients respond to symptoms with 2 predominant activity patterns—activity limitation and all-or-nothing behaviors—both of which may contribute to illness persistence. The current study investigated whether activity patterns occurred at the same time as, or followed on from, patient symptom experience and affect. ***Method:*** Twenty-three adults with CFS were recruited from U.K. CFS services. Experience sampling methodology (ESM) was used to assess fluctuations in patient symptom experience, affect, and activity management patterns over 10 assessments per day for a total of 6 days. Assessments were conducted within patients’ daily life and were delivered through an app on touchscreen Android mobile phones. Multilevel model analyses were conducted to examine the role of self-reported patient fatigue, pain, and affect as predictors of change in activity patterns at the same and subsequent assessment. ***Results:*** Current experience of fatigue-related symptoms and pain predicted higher patient activity limitation at the current and subsequent assessments whereas subjective wellness predicted higher all-or-nothing behavior at both times. Current pain predicted less all-or-nothing behavior at the subsequent assessment. In contrast to hypotheses, current positive affect was predictive of current activity limitation whereas current negative affect was predictive of current all-or-nothing behavior. Both activity patterns varied at the momentary level. ***Conclusions:*** Patient symptom experiences appear to be driving patient activity management patterns in line with the cognitive–behavioral model of CFS. ESM offers a useful method for examining multiple interacting variables within the context of patients’ daily life.

Chronic fatigue syndrome (CFS) is characterized by the experience of persistent and severe fatigue in addition to other symptoms such as pain, sleep disturbance, and reported cognitive deficits (Fukuda et al., 1994). Cognitive–behavioral models propose that patient cognitions and behaviors interact in a complex fashion with patient symptom experience and affect in the perpetuation of CFS (Chalder, Tong, & Deary, 2002; Deary, Chalder, & Sharpe, 2007; Surawy, Hackmann, Hawton, & Sharpe, 1995). It is suggested that patients’ beliefs about their symptoms (e.g., that they are indicative of damage) and about appropriate responses to symptoms (e.g., that they should avoid activity to avoid exacerbating symptoms) drive their symptom management behavior (Knoop, Prins, Moss-Morris, & Bleijenberg, 2010). Focusing on symptoms, catastrophizing about symptoms, and the belief that symptoms mean harm are suggested to lead to two predominant forms of behavioral response to symptoms—“all-or-nothing behavior” and “activity limitation”—which themselves contribute to dysregulation and the maintenance of symptoms (Moss-Morris, 2005).

There is evidence that all-or-nothing behavior, in which bursts of intense activity when feeling relatively well are interspersed with periods of extended rest in response to symptoms, is associated with the initial persistence of fatigue symptoms and onset of CFS after glandular fever (Moss-Morris, Spence, & Hou, 2011). There is less evidence that all-or-nothing behavior is involved in the maintenance of symptoms, although reduced all-or-nothing behavior did mediate a small proportion of the effects of cognitive behavior therapy (CBT) and graded exercise therapy on fatigue in one study (Cella, White, Sharpe, & Chalder, 2013; Chalder, Goldsmith, White, Sharpe, & Pickles, 2015). Therefore, all-or-nothing behavior is regarded as a potentially unhelpful management strategy.

Patient avoidance of activity has been shown to be directly linked with patient beliefs about the physical origin of symptoms, increased fatigue severity (Vercoulen et al., 1998), patient beliefs about pain, and increased pain intensity (Nijs, Van de Putte, Louckx, Truijen, & De Meirleir, 2008). There is evidence to suggest that pain and fatigue decrease simultaneously in response to treatment (Bloot, Heins, Donders, Bleijenberg, & Knoop, 2015; Bourke, Johnson, Sharpe, Chalder, & White, 2014; Knoop, Stulemeijer, Prins, van der Meer, & Bleijenberg, 2007), although the dynamic relationship among pain, fatigue, and other perpetuating variables is not currently well understood (Nijs et al., 2012). Evidence for the role of avoidance or activity limitation in the maintenance of fatigue comes from treatment studies. Reduced self-reported activity limitation has been shown to mediate improvement in fatigue symptoms after treatment (Heins, Knoop, Burk, & Bleijenberg, 2013; Wearden & Emsley, 2013), and change in the beliefs underlying activity limitation (“fear avoidance beliefs”) mediates change in fatigue after cognitive–behavioral and graded exercise treatment (Chalder et al., 2015). Therefore, there is some evidence to suggest that over time, activity patterns are associated with perpetuation of symptoms, and that, with treatment, change in activity patterns are associated with reduction in fatigue. However, little is known about what initiates and maintains these activity patterns on a moment-to-moment or day-to-day basis. For example, it is not known whether, during the course of a day, patients rest in response to symptoms, are intensely active when they are feeling well (i.e., all-or-nothing behavior), or avoid activity more generally over the course of the day.

Therefore, the present study aimed to concurrently examine aspects of the cognitive–behavioral model to further understanding of the factors predicting patient activity patterns in CFS. A mobile phone-based app (Ainsworth et al., 2013) was used to examine interrelationships between short-term fluctuations in fatigue-related symptoms, pain, and affect (positive and negative) and concomitant and subsequent activity patterns.

## Hypotheses

It was hypothesized that higher fatigue-related symptom reporting would be associated with self-reported activity limitation at the current assessment and at the subsequent assessment; that is, that participants would demonstrate more activity limitation in response to fatigue. To assess the independent contribution of pain in driving patient activity management strategies, activity limitation and all-or-nothing behavior were examined in association with pain. Higher pain was predicted to be associated with higher activity limitation and lower all-or-nothing behavior at current and subsequent assessments. In line with cognitive–behavioral models, it was also predicted that higher negative affect and lower positive affect would be associated with activity limitation. It was hypothesized that subjectively feeling well would be associated with self-reported all-or-nothing behavior at the current assessment and at the subsequent lagged assessment; that is, the participant would report higher activity when they were feeling well. In addition, it was predicted that higher levels of positive affect and lower levels of negative affect would be associated with more all-or-nothing behavior.

## Method

### Participants

Participants with a clinical diagnosis of CFS/ME (Chronic fatigue syndrome/ Myalgic encephalomyelitis) were recruited from specialist U.K. National Health Service (NHS) CFS/ME services; the final sample included 23 patients ranging from 17 to 58 years old, with a mean age of 35.5 (*SD* = 13.96) years. Upon entry in the study, patients had been ill for a median of 5 years (interquartile range [IQR] = 10) and had recently been enrolled in specialist treatment programs, delivering either CBT based on the cognitive–behavioral model, or pragmatic rehabilitation, a therapy that combines elements of CBT and graded exercise therapy (Wearden et al., 2010).

### Procedure

Participants were loaned an Android smartphone with a modified CFS-specific version of the Clintouch app (Ainsworth et al., 2013) installed. A standard experience sampling methodology (ESM) protocol was followed (Myin-Germeys, Delespaul, & van Os, 2003) in which participants received 10 assessments per day for a period of 6 days. The assessments were signaled by an alert, and they were scheduled according to an identical semirandom schedule for all participants. One assessment occurred within each 90-min period throughout the day between 7:30 a.m. and 10:30 p.m.; the time elapsed between assessments ranged from 29 to 162 min (*M* = 88.52, *SD* = 34.03 min). Participants were instructed that an alert would signal a momentary assessment and that there would be a 15-min period in which to begin the assessment before the questions expired.

### Measures

All items were measured on a momentary basis (i.e., “Before the beep went off I was . . .” or “Right now I am . . .”) and were rated on a 7-point Likert scale anchored from 1 (*not at all*) to 7 (*a lot*).

#### Patient activity management (cognitive–behavioral) strategies

Items assessing patient activity management strategies were modified for ESM from the Cognitive-Behavioral Response Questionnaire (Skerrett & Moss-Morris, 2006). Activity limitation was assessed by two items: “resting to control my symptoms” and “avoiding activities that might make my symptoms worse” (α = .80 for these items). Two further items were included to assess all-or-nothing behaviors. These items were “rushing to get things done while I feel able” and “doing things while I can” and loaded on to a single-factor solution (α = .87).

#### Patient affect

Standard ESM affect items were used to assess patient affect (Myin-Germeys et al., 2003). Positive affect was assessed by five items: excited, happy, satisfied, relaxed, and cheerful (α = .87). A further five items were included to assess negative affect, including sad, annoyed, irritated, anxious, lonely, and guilty (α = .87).

#### Symptoms

Symptom items were adapted from the well-validated Chalder Fatigue Questionnaire (Chalder et al., 1993). Four items were used to assess fatigue-related symptom severity in the moment. These included feeling weak, tired, experiencing mental fog, and being sleepy (α = .73). Patient pain was assessed by a single item relating to the extent to which pain was being experienced in the moment. It is recommended that positively and negatively phrased items are included within ESM assessments (Palmier-Claus et al., 2011), and previous exploration of patient daily experiences of living with a chronic condition has suggested that feeling “well” is not simply an absence of symptom experience (Olsson, Skär, & Söderberg, 2010). Therefore, to assess the extent to which they felt “well” in the moment, two items, “feeling well” and “feeling active,” were included (α = .74).

### Participant Compliance

A total of 1,380 assessments were delivered across the sample, and of these, 893 were initiated within 15 min of an alert (65% compliance). Participants completed between 15 and 60 assessments (*M* = 38.83, *SD* = 14.83). The average number of daily assessments completed by participants was 6.47. Traditionally, participants who complete fewer than 20 momentary assessments are excluded from analyses (Palmier-Claus et al., 2011); three participants within the current sample completed 15, 15, and 16 assessments, respectively. Preliminary analyses were conducted excluding these participants. However, to exploit all of the available data, all of the participants were retained in the final analyses.

### Statistical Analysis

Multilevel models were used to examine study hypotheses, taking into account the hierarchal structure of ESM data. The XTMIXED command was used in Stata (StataCorp, 2009) for all continuous outcome variables, with a random intercept for each participant and for each day within participant; β, 95% confidence interval (CI), and *p* values are reported for all associations between independent and dependent variables. Predictor variables included patient affect and symptoms at the same (*t*) and previous (*t* − 1) assessment. These were grand mean centered before inclusion as predictor variables in all models. Patient activity management strategies were included as the dependent variables (*t*). Intraclass correlation coefficients (ICCs) were calculated for each of the predictor variables to enable the proportion of variability in each level of the data (i.e., assessment, day, and person levels) to be explored.

## Results

### Predicting Patient Activity Limitation

As predicted, patient symptom severity was associated with increased self-reported activity limitation at the concomitant and subsequent assessments (see Table 1). In addition, higher levels of current and previous pain predicted increased activity limitation at the current assessment. In contrast to study hypotheses, patient-reported negative affect did not significantly predict patient activity limitation on a momentary basis. Greater patient-reported positive affect approached significance in predicting increased activity limitation at the current assessment (*p* = .056); the direction of this relationship was opposite to that hypothesized.[Table-anchor tbl1]

### Predicting All-or-Nothing Behaviors

Patient reports of feeling well were associated with higher levels of reported all-or-nothing behaviors at the current assessment (see Table 1). In addition, feeling well at the current assessment significantly predicted increased all-or-nothing behavior at the subsequent assessment. Higher levels of pain at the current assessment did not predict all-or-nothing behavior at the current assessment but were predictive of less all-or-nothing behavior reported at the subsequent assessment. Patient reports of positive affect were not found to significantly predict patient all-or-nothing behavior, and in further contrast to study hypotheses, higher negative affect predicted more all-or-nothing behavior at the current assessment.

### The Variability of Activity Management Strategies Across the Different Levels of Data

When examining the ICC analyses, it was identified that the majority of the unexplained variation in patient activity management strategies was at the current assessment level for all predictor variables (see Table 1). For example, all-or-nothing responses showed 82% variance from one assessment to another, within the same patient and across the same day when feeling well was the predictor variable, whereas 15% of the variance was due to differences between individual participants.

## Discussion

This study aimed to examine the interrelations between short-term fluctuations in patient self-reported fatigue-related symptoms, pain, and affect and concomitant and subsequent activity patterns in CFS. The main findings show that patient activity patterns arise in response to patient symptom experience, the effects of which were found to extend beyond the immediate context in which the symptoms were being experienced. In line with study hypotheses, patients reported limiting their activity more (i.e., resting) when they were experiencing higher levels of fatigue-related symptoms and higher pain. In addition, patients reported more all-or-nothing type activity strategies when they were feeling subjectively well and less after high levels of pain. These results support the cognitive–behavioral maintenance model for CFS (Chalder et al., 2002; Deary et al., 2007; Surawy et al., 1995) by demonstrating that patient activity management is, at least to some extent, driven by symptom experiences. Patients in our study had recently been enrolled into a course of either CBT or pragmatic rehabilitation. Over time, both of these treatments will help patients to understand that activity limitation and all-or-nothing behavior are not helpful responses to symptoms, and both will encourage a gradual, programmed increase in activity levels based on collaboratively agreed goals rather than driven by symptoms. It is a limitation of the study that the exact amount of treatment each patient had received, if any, was not recorded at the time of completing the ESM measures. However, because patients participated either before or early in the course of their treatment, it is likely that they had not yet started to benefit from the changes in beliefs and behavior that treatment would be expected to bring about. Cognitive–behavioral and graded-exercise therapies are recommended treatments for CFS in the United Kingdom (National Institute for Health and Care Excellence, 2007) and demonstrate small to moderate effects in improving patient illness outcomes (Castell, Kazantzis, & Moss-Morris, 2011; White et al., 2011). Both of these therapies involve a gradual and programmed increases in activity and may result in breaking the link between experiencing symptoms and activity levels, thus modifying patient beliefs and behavioral responses that are thought to perpetuate CFS (Moss-Morris et al., 2013).

Cognitive–behavioral maintenance models for CFS have previously been criticized for lacking specificity (Knoop et al., 2010), with evidence accumulated for the role of individual perpetuating factors in isolation (Moss-Morris, 2005). Little empirical research has focused on the interaction of several factors thought to be important in symptom perpetuation and maintenance (Deary et al., 2007). By using ESM to facilitate data collection, the effect of both symptom experience, including fatigue-related symptoms, and pain could be examined simultaneously alongside patient affect in predicting activity management patterns. The current results indicate that activity limitation was predicted by pain and fatigue-related symptoms, suggesting that to develop theory and understanding of these complex processes, the relationship among pain, fatigue, and patient cognitive–behavioral variables needs further investigation (Nijs et al., 2012). It is interesting to note that the analyses indicate that at a momentary level at least, patient affect is predictive of an activity pattern opposite to that originally hypothesized. This finding was applicable to activity limitation and all-or-nothing responses because, contrary to our predictions, higher levels of positive affect were significantly associated with activity limitation and higher levels of negative affect were associated with all-or-nothing behavior. Although this at first sight seems counterintuitive, we speculate that these findings may reflect patient beliefs about the meaning of the relationship between symptoms and activity management strategies. The association among positive affect, fatigue-related symptom severity, pain, and activity limitation may potentially reflect underlying patient beliefs that limiting activity (i.e., resting) is a beneficial strategy for coping with increased symptom severity or pain. Likewise, it is possible that feeling well may provide patients with an opportunity to engage in increased activity but may be accompanied by patient beliefs that subsequent worsening symptoms are inevitable, beliefs that may then be associated with negative affect.

However, the limitations associated with some of the items included within the current study must be acknowledged. First, pain was assessed by a single item at each assessment, and although fatigue-related severity was assessed using four items relating to common experiences of fatigue, fatigue was not included as an item. Second, those items relating to activity limitation required patients to report on their behavior (e.g., are they limiting activity in that moment) and make a judgment relating to that behavior (e.g., is this to control their symptoms), thereby confounding beliefs about symptoms and symptom management with reports of activity. This was a design flaw of the study, which arose because, to provide some comparability with other studies, items from the cognitive–behavioral response questionnaire (a measure of patient activity management) were used. Future studies would benefit from including objective measures of activity, which are separate from measures of patient beliefs about activity. Including pure activity measures would assist us to further develop a theoretical understanding of the dynamic relationships between symptoms and activity. In addition, utilization of mobile-health capabilities, such as incorporating ESM studies alongside established treatment programs, would also enable assessment of the potential mechanisms of change during treatment (Ritterband, Thorndike, Cox, Kovatchev, & Gonder-Frederick, 2009). For example, it would be possible to examine whether hypothesized changes in cognitions are responsible for changes in activity management behaviors (Knoop et al., 2010).

Our findings demonstrated that both all-or-nothing behavior and activity limitation varied most at the individual assessment level, indicating that patients were reporting high variability in the extent to which they were engaging in both activity patterns across assessments within the course of each day. In contrast, the results indicated little variation in the activity management patterns between different days (within the same person), with some variation observed between individual patients. These findings are in line with previous findings demonstrating that objective activity levels in CFS patients are also variable at the individual level (Evering, Tonis, & Vollenbroek-Hutten, 2011). Furthermore, the results indicate that symptom severity, pain, and affect are independently predicting activity management strategies across different time frames, although it is important to note that the time between assessments varied across the study, from approximately 30 to 160 min. Our study drew upon previous ESM sampling schedules in designing the frequency of the assessments (Myin-Germeys et al., 2003), but this could be further strengthened by taking account of the advances in mobile health, for example, by using sensing capabilities to prompt individualized, tailored assessments in real time (Spruijt-Metz & Nilsen, 2014). Combining this with clearly defined cognitive–behavioral components could form the basis for building an interactive digital intervention (Nahum-Shani et al., 2015) to help patients to understand their activity patterns and disassociate activity from symptom experiences or affect. For example, in response to current reports of symptom severity, such an intervention might guide patients to address unhelpful activity beliefs using cognitive–behavioral strategies (White et al., 2011) before they engage in prolonged periods of activity limitation. Likewise, prompts could be delivered to engage patients in graded activities in accordance with a predetermined, agreed, and acceptable activity schedule, with additional alerts and reminders programmed using algorithms for all-or-nothing or activity limitation type activity patterns.

### Conclusions

The current findings suggest that two unhelpful activity management patterns in CFS arise as a result of patient symptom experience and affect. Although the results reported here must be interpreted tentatively given the small sample size; combining ESM with mobile health enabled us to demonstrate that it is feasible to examine these complex associations between known perpetuating factors in CFS in the context of daily life. In further developing a complex understanding of the interrelations between these variables, it may be possible to pinpoint when the unhelpful behavioral patterns begin. Future studies may utilize m-health capabilities to not only develop theoretical understanding of maintenance of CFS symptoms, but to work toward interactive digital interventions to enact symptom improvement.

## Figures and Tables

**Table 1 tbl1:** The Association Among Patient Affect, Symptom Experience Variables, and Cognitive-Behavioral Strategies in Current (t) and Lagged (t − 1) Analyses, and the ICCs for Individual Patient Predictor Variables

Predictor variables	Activity limitation			ICC		
	β	*SE*	*p*	Person	Day	Beep
Symptom severity						
Current	**.155**	**.013**	**<.001**	.09	.08	.83
Lagged (*t* − 1)	**.030**	**.009**	**.001**	.15	.08	.77
Pain						
Current	**.298**	**.046**	**<.001**	.09	.06	.85
Lagged (*t* − 1)	**.310**	**.052**	**<.001**	.10	.09	.81
Negative affect						
Current	.043	.065	.513	.17	.08	.75
Lagged (*t* − 1)	.038	.075	.610	.16	.09	.75
Positive affect						
Current	.102	.054	.056	.18	.08	.74
Lagged (*t* − 1)	.011	.062	.863	.16	.09	.75
	All-or-nothing behavior			ICC		
	β	*SE*	*p*	Person	Day	Beep
Feeling well						
Current	**.700**	**.025**	**<.001**	.14	.03	.83
Lagged (*t* − 1)	.218	.033	**<.001**	.15	.03	.82
Pain						
Current	.023	.084	.784	.12	.05	.83
Lagged (*t* − 1)	**−.252**	**.094**	**.008**	.15	.02	.83
Positive affect						
Current	.152	.094	.104	.12	.05	.73
Lagged (*t* − 1)	.032	.107	.762	.14	.02	.84
Negative affect						
Current	**.312**	**.113**	**.006**	.11	.06	.83
Lagged (*t* − 1)	.090	.127	.480	.14	.02	.84
*Note*. ICC = Intraclass correlation coefficients. *p* < .05 is in bold.						
